# Cryotherapy Reduced Postoperative Pain in Gynecologic Surgery: A Randomized Controlled Trial

**DOI:** 10.1155/2019/2405159

**Published:** 2019-03-04

**Authors:** Apisada Chumkam, Densak Pongrojpaw, Athita Chanthasenanont, Junya Pattaraarchachai, Kornkarn Bhamarapravatana, Komsun Suwannarurk

**Affiliations:** ^1^Department of Obstetrics and Gynecology, Faculty of Medicine, Thammasat University, Pathum Thani, Thailand; ^2^Chulabhorn International College of Medicine, Thammasat University, Pathum Thani, Thailand; ^3^Department of Preclinical Science, Faculty of Medicine, Thammasat University, Pathum Thani, Thailand

## Abstract

**Objective:**

To examine the effectiveness of cryotherapy for reducing postoperative pain in patients who underwent exploratory laparotomy for gynecologic surgery.

**Materials and Methods:**

Patients who had indication for an exploratory laparotomy gynecologic procedure were selected by attending physicians to undergo abdominal surgery via low transverse skin incision. The participants were randomized into study and control groups with simple random sampling methods. Cold packs were applied at two hours after operation for 6 hours. The visual analog scale (VAS) score was recorded at two, 6, and 12 hours after operation.

**Result:**

One hundred cases were recruited and then divided into study and control groups equally. The mean age of both groups was 43 years. There was no difference in demographics data of both groups. Half of the participants in both groups underwent hysterectomies. At two hours after surgery, both groups had similar VAS scores. The study group had a lower VAS score at 6 and 12 hours after surgery than the control group with statistical difference. Morphine consumption within 24 hours after surgery in both the study and control groups was 2.8±3.4 and 3.0±4.4 mg, respectively, with no statistical difference. However the registration time of the first morphine requirement in the study group was statistically more prolonged than that of the control group. The lengths of hospital stay in both groups were similar. There was no complication reported in this study.

**Conclusion:**

Cryotherapy can reduce postoperative pain. In this presented study the patients who underwent gynecologic surgery had improved pain relief and prolonged time for the first dose of the analgesic drug.

## 1. Introduction

Postoperative pain is a major concern of many patients. It is inadequately treated in as many as 30 to 50% of all postoperative patients [1]. Tissue injury is produced by surgery consequently by release of histamine and inflammatory mediators. They both induced vasodilation and extravasation. Inflammatory mediators activate the noxious stimuli pathway, which is then transduced by peripheral nociceptors. Nerve impulse is transmitted from peripheral visceral and somatic sites to the dorsal horn of the spinal cord, where integration of peripheral nociceptive and descending modulatory input occurs [2].

When patients' postoperative pain decreased, patients reported less complications, i.e., no atelectasis and bowel dysfunction, and less side effects from pain relievers. The most common medications given for postoperative pain are opioids and opioid derivatives. They are known to cause dizziness, sedation, nausea, vomiting, physical dependence, tolerance, and respiratory depression [1].

Cryotherapy is a popular nonpharmacological intervention used to relieve pain following musculoskeletal injury, major surgical procedures, and vaginal delivery [3–9]. Furthermore, cryotherapy has many positive physiologic effects, namely, reduction in blood flow, edema, hemorrhage, enzyme activity, and tissue damage. Cryotherapy was shown to increase the pain threshold and tolerance by reducing nerve conduction velocity and muscle spasm especially when the temperature was lower than 80.6°F (27°C) [6].

Many clinical studies studied the effectiveness of cryotherapy in postoperative pain reduction [3–9]. In a randomized control trial setting by Walkins, ice packs were used for pain relief after abdominal surgery via midline incision. The use of an ice pack showed significant reduction in postoperative pain at one and three days after surgery and decreased morphine consumption within 24 hours after surgery [3].

More than 80% of the patients that underwent gynecological operations experienced severe pain [10]. Furthermore, in order to avoid inducing or aggravating pain, patients might hold in their coughing and deep breathing. This act might cause predisposition to atelectasis development. Patients with postoperative pain might also minimize position change in bed and refuse ambulation. Postoperative immobilization in patients could result in platelet adhesion increase, development of deep vein thrombosis (DVT), and pulmonary embolism [11].

The aim of this study was to compare the effectiveness of cryotherapy to that of standard postoperative pain management in gynecologic surgery with low transverse incision.

## 2. Materials and Methods

A randomized controlled study was conducted at Thammasat University Hospital, Pathum Thani, Thailand, to compare the effectiveness of cryotherapy and that of standard postoperative pain treatment after gynecologic surgery. Approval was obtained from the Human Ethics Committee of Thammasat University (MTU-EC-OB-1-192/60) and registered with the Thai Clinical Trials Registry (TCTR20180226004). Patients with low transverse incision would be assigned cold pack compressions at the skin incision site compared to standard postoperative care.

Participants were gynecologic patients aged 15 years or more who had indication for an exploratory laparotomy procedure. They were selected by attending physicians to perform low transverse line incision between March and September 2018. Patients were excluded if there were contraindication to cold therapy, surgical complication, or extended incision to the low midline, local anesthesia or epidural block was used, they had undergone repeated exploratory laparotomy within 24 hours postoperatively, and they declined to participate in the study.

Patients were approached by gynecologic physicians at the day of their hospital admission about participating in the study. Inclusion criteria were confirmed before obtaining any written consent. The participants were randomized into two groups of treatment with simple random sampling methods. Patients' demographics data included age, weight, height, occupation, education, income, underlying disease, parity, menopausal status, diagnosis in this admission, surgical incision, and operation. Group's allocation was concealed in sealed packed opaque envelopes that were opened after the completion of the surgery. Surgeon was blinded to assigned treatment group before the intervention started.

Both groups received standard postoperative care. It consisted of vital sign recording, an analgesic agent, an antibiotic, Foley's catheter retention, surgical wound dressing with a waterproof transparent patch, and maintenance hydration. Participants in the treatment group received cold pack gel (Siriraj Jelly Cold-Hot Pack, Thailand) compression treatment. The cold pack in its own thin cloth bag was kept at -4°C ready for future use. The cold pack was then placed on top of the surgical wound at two hours after surgery. It was changed every two hours for two consecutive times. Patients were evaluated for complications of cryotherapy by verbal interviews at 30 and 60 minutes. Any complaint would be recorded. Patients were asked about their pain scores every two hours. The control group received standard postoperative care [11].

Intravenous 3 mg morphine was given to any patients from both groups if their reported pain scores were equal to or greater than 6. Amount of morphine consumption and its possible side effects, namely, nausea, vomiting, itching, respiratory depression, and allergic reaction, were also recorded in the first 24 hours after operation.

The sample size in this study was calculated from the standard deviation of the control group (SD = 2.89) from a pilot study (between September and October 2017 with a total of 20 patients). The alpha and beta were set at 0.01 and 0.05, respectively. A sample size of 100 patients (50 patients per group) was obtained from the calculation.

The primary outcome was the visual analog scale (VAS) score at 6 and 12 hours after surgery. The starting time was defined as the time when incised skin was completely sutured. The VAS was ranked from score 0 (no pain) to 10 (worst pain). Secondary outcomes were morphine consumption within 24 hours after surgery, time for the first dose of the analgesic drug administered, length of hospital stay, and surgical site infection rate.

Data were analyzed by using the Statistical Package for the Social Sciences (SPSS Inc., Chicago, IL, USA) for Windows, version 24. Continuous data were represented by mean and standard deviation. The VAS score was analyzed by repeated ANOVA. Categorized data were evaluated by the chi-square tests or Fisher exact test whichever appropriated. The level of statistical significance was set at a* p* value less than 0.05.

## 3. Results

**Figure 1:**
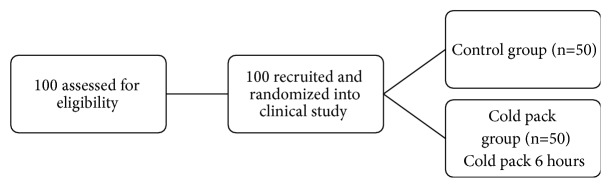
Participants flow diagram.

One hundred women were enrolled into the study and control groups. Each group was composed of 50 cases (Figure 1). There was no difference in the demographics data of both groups including age, weight, height, underlying diseases, parity, and menopausal status (Table 1). Both groups had no significant difference in preoperative diagnosis, type of operation, and incision length (Table 2).

**Figure 2:**
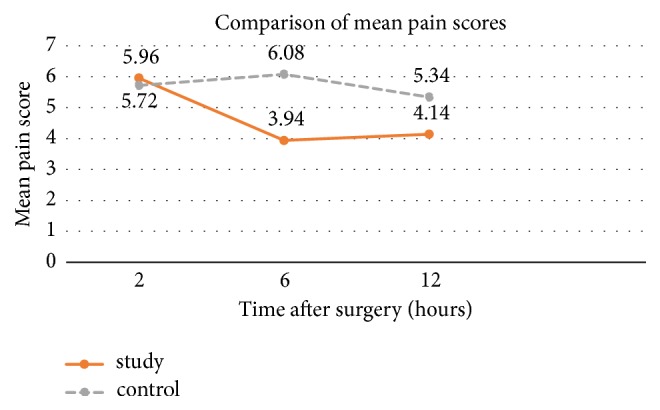
Comparison of mean postoperative pain scores (VAS).

Length of transverse surgical incision in both groups regardless of their procedures was around 13 cm without statistical significance (Table 2). At two hours after surgery, both groups had similar VAS scores. The study group had lower VAS scores at 6 and 12 hours after surgery than the control group with statistical difference as represented in Table 3.  Figure 2 represented the comparison of mean postoperative pain scores (VAS) as a line chart.

**Figure 3:**
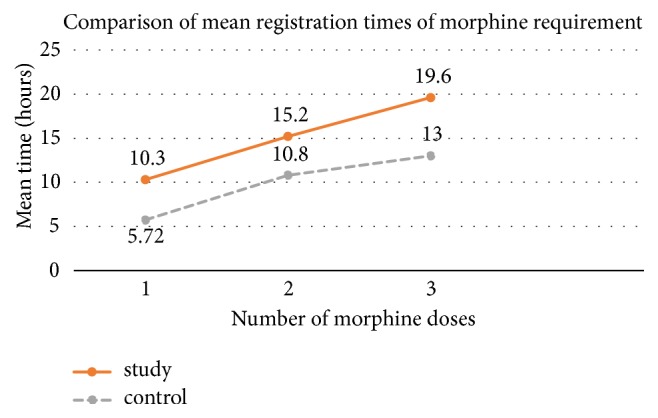
Comparison of mean registration times of morphine requirement.

Morphine consumption within 24 hours after surgery in both the study and control groups was 2.8±3.4 and 3.0±4.4 mg, respectively, with no statistical difference. However the registration time of the first morphine dose requested in the study group was statistically longer than that of the control group (10.2±4.5 and 7.2±3.6 hours, respectively) as presented in Figure 3. The registration times of the second and third morphine dose requested were not statistically different (Table 3). The lengths of hospital stay in both groups were similar (Table 3). There was no complication report in this study.

## 4. Discussion 

Cryotherapy for pain relief has been used for many years in the treatment of localized tissue trauma. The temperature reduction of the soft tissue by 10 to 15°C was reported to decrease local metabolism and oxygen requirement. Soft tissue temperature reduction also induces peripheral blood vessels constriction, resulting in the reduction of tissue swelling, bleeding, bruising, and localized pain [5].

In the present study, cryotherapy reduced postoperative pain in patients who underwent gynecologic surgery at 6 and 12 hours compared to the control group. Cryotherapy in our study was an applied cold pack to the surgical site for 6 consecutive hours after operation. Its effect lasted long until 12 hours after surgery.

There are many clinical studies that studied the effectiveness of cryotherapy [3–9]. The results of the studies showed cryotherapy might reduce postoperative pain as represented in Table 4. Koç and coworkers used ice packs to reduce postoperative pain in patients who underwent inguinal hernia repair with standard general anesthesia compared to patients without ice pack treatment. They used the VAS to evaluate postoperative pain in both groups. There were significant differences in pain relief 2 and 6 hours after surgery (*p*<0.05) [7]. Their results and our report showed the same cryotherapy result for as long as 6 hours after surgery even though these were different operations (hernia versus gynecologic surgery). It seemed that the cryotherapy benefit for postsurgery treatment could be seen in a broad spectrum manner (Table 4).

Wanlayanee and coworkers studied 28 patients who underwent benign gynecologic surgery. Gel packs were applied at two hours after operation for 20 minutes. Gel pack application showed statistically significant result in postoperative pain reduction when pain was divided into moderate to severe and mild pain intensity at 6 hours after surgery. Their patients who had moderate to severe pain in the control and study group were 11 and 8 (*p*<0.05) but there was no difference in the mild pain score. Opioid consumption, hospital stay, and wound infection showed no statistical difference between both groups [4]. In the present study, a large number of participants were recruited. The extended time of cold pack use after surgery was as up to 6 hours. We found that the cold pack compression could effectively reduce postoperative pain up to 12 hours after surgery.

The mean morphine consumption within 24 hours after surgery in the control and study groups was 3.02±4.52 and 2.8±3.35 mg, respectively, without statistical significance. The amount of morphine consumption was not a primary objective of this current study.

The strength of our study was that it was a randomized controlled trial and the surgeons were blind to the assigned treatment groups. Multiple primary and secondary outcomes measured were obtained for comparative analysis. Our limitation was the unblinded intervention.

## 5. Conclusion

Cryotherapy can reduce postoperative pain. In this presented study patients who underwent gynecologic surgery had improved pain relief and prolonged time for the first dose of the analgesic drug. The use of cryotherapy can improve postoperative pain control. Cryotherapy, in our opinion, should be widely used in postoperative pain control because it is a noninvasive mode of operation with a very effective cost, no complication, and a very favorable patient report.

## Figures and Tables

**Table 1:** Demographic characteristics of participants (50 cases per group).

	Control	Study	*p* value
Age (years)*∗*	43±11.21	42.88±13.13	0.961
Weight (kg)**∗**	58.86±10.73	58.28±10.56	0.786
Height (cm)*∗*	157.92±4.15	157.44±4.39	0.576
Education level*∗ ***∗**			0.304
Primary school	2 (4)	6 (12)	
High school	13 (26)	19 (38)	
Bachelor's degree	32 (64)	23 (46)	
Higher than Bachelor's degree	3 (6)	2 (4)	
Occupation*∗∗*			0.254
Agriculture	6 (12)	9 (18)	
Self-employed	19 (38)	22 (44)	
Employee	10 (20)	11 (22)	
Government officer	11 (22)	8 (16)	
Others^a^	4 (8)	0 (0)	
Income (baht)*∗∗*			0.789
<10,000	4 (8)	3 (6)	
10,000-30,000	23 (46)	21 (42)	
30,000-50,000	19 (38)	19 (38)	
>50,000	4 (8)	7 (14)	
Underlying disease*∗∗*			0.212
None	40 (80)	38 (76)	
Hypertension	2 (4)	7 (14)	
Dyslipidemia	2 (4)	2 (4)	
Others^b^	5 (10)	3 (6)	

*∗*: mean ± standard deviation (SD); *∗∗*: n(%); others^a^: housewife, student; others^b^: diabetes mellitus, cardiovascular disease, anemia, coagulopathy, hypothyroid, or hyperthyroid.

**Table 2:** Gynecologic history of participants (50 cases per group).

	Control	Study	*p* value
Hormone used*∗∗*	4 (8)	10 (20)	0.084
Surgery*∗∗*	5 (10)	6 (12)	0.749
Parity*∗*	1.16±1.4	1.20±1.36	0.885
Menopausal status*∗∗*			0.79
Premenopausal	41 (82)	42 (84)	
Postmenopausal	9 (18)	8 (16)	
Diagnosis*∗∗*			0.184
Myoma uteri	15 (30)	12 (24)	
Ovarian cyst	14 (28)	12 (24)	
Endometriosis	5 (10)	5 (10)	
Adenomyosis	6 (12)	2 (4)	
Ectopic pregnancy	2 (4)	2 (4)	
Molar pregnancy	1 (2)	1 (2)	
Endometrial hyperplasia	2 (4)	0 (0)	
Tuboovarian abscess	0 (0)	2 (4)	
Others^c^	5 (10)	14 (28)	
Type*∗∗*			0.435
Maylard incision	13 (26)	17 (34)	
Pfannenstiel incision	36 (72)	33 (66)	
Cherney incision	1 (2)	0 (0)	
Length*∗*	13.22±2.47	13.84±3.13	0.274
Procedure*∗∗*			0.775
TAH with BSO	15 (30)	13 (26)	
TAH	6 (12)	6 (12)	
SO	8 (16)	6 (12)	
Cystectomy	9 (18)	7 (14)	
Myomectomy	5 (10)	4 (8)	
Others^d^	7 (14)	14 (28)	

*∗*: mean ± standard deviation (SD); *∗∗*: n(%); hormone used: history of hormone used; surgery: history of surgery; history of STD: history of sexual transmitted disease; others^c^: cervical cancer, endometrial cancer, ovarian cancer, and breast cancer; type: type of incision; length: length of incision; TAH with BSO: transabdominal hysterectomy with bilateral oophorectomy; TAH: transabdominal hysterectomy; SO: salpingo-oophorectomy; others^d^: surgical staging, salpingectomy, and salpingostomy.

**Table 3:** Comparison of primary outcome and secondary outcome (50 cases per group).

	Control	Study	*p* value
Primary outcome (VAS) *∗*			
2	5.72±2.25	5.96±2.55	0.619
6	6.08±2.29	3.94±1.89	<0.005
12	5.34±2.19	4.14±1.89	<0.005
Secondary outcome*∗*			
Morphine consumption	3.32±4.52	2.8±3.35	0.515
Time for 1^st^ dose of morphine	7.17±3.63	10.21±4.52	0.012
Time for 2^nd^ dose of morphine	10.78±3.47	15.17±4.24	0.463
Time for 3^rd^ dose of morphine	13±1.49	19.67±3.51	0.102
Length of hospital stay	4.74±1.28	4.86±1.28	0.639

*∗*: mean ± standard deviation (SD); VAS: visual analog scale score; 2: at 2 hours after procedure; 6: at 6 hours after procedure; 12;: at 12 hours after procedure.

**Table 4:** Comparing of cold pack efficacies.

Research	Koç	Watkins	Rotenburg	Wanlayanee	Present
Years	2006	2013	2013	2018	2018
Country	Turkey	USA	Canada	Thailand	Thailand
Operation	Hernia	Sx	Tonsil	Benign gyn	Gyn
Type	Lichtenstein	Mid	Excision	Mid+LT	LT
Duration	20 min	24 hr	24 hr	20 min	6 hr
Number	40	55	18	25	100
Result	IE	IE	IE	IE	IE
LOS				ND	ND
Analgesic	D	D		ND	ND

Time: time for started treatment; hr: hours; min: minutes; hernia: herniorrhaphy; Sx: general surgery; tonsil: tonsillectomy; gyn: gynecologic surgery; Lichtenstein: Lichtenstein type repair; mid: midline incision; duration: duration of treatment; LT: low transverse incision; LOS: length of hospital stay; analgesic: analgesic consumption; IE: increases efficacy: ND: no difference; D: decreased consumption.

## Data Availability

The datasets generated and analyzed during the current study are available from the corresponding author upon request.
